# The impact of an online intervention on the medical, dental and health sciences students about interprofessional education; a quasi-experimental study

**DOI:** 10.1186/s12909-021-02900-8

**Published:** 2021-08-30

**Authors:** Salman Y. Guraya, Leena R. David, Shermin Hashir, Noha A. Mousa, Saad Wahbi Al Bayatti, Ahmed Hasswan, Mujtaba Kaouas

**Affiliations:** 1 College of Medicine, University of Sharjah, Sharjah, United Arab Emirates; 2 College of Health Sciences, University of Sharjah, Sharjah, United Arab Emirates; 3 College of Dental Medicine, University of Sharjah, Sharjah, United Arab Emirates

**Keywords:** Interprofessional education, Teamwork, Medical students, Intervention, Online

## Abstract

**Background:**

Interprofessional education (IPE) encompasses integration, communication, mutual trust and shared decision-making with a common goal of improved patient care and safety. Despite its crucial role, IPE has not gained its anticipated popularity. This study aims to determine the impact of an online educational intervention about IPE on medical, dental and health sciences students in the University of Sharjah (UoS).

**Methods:**

This quasi-experimental research was conducted in three phases; a pre-intervention phase where the Readiness for Interprofessional Learning Scale (RIPLS) inventory was administered online to the medical, dental and health sciences students of UoS; an intervention phase where an online workshop was organized via Microsoft Teams®; and a post-intervention phase where RIPLS was used to gather the students’ attitudes towards IPE. The independent *t* test was used to compare the responses between genders and junior and senior students. A paired sample *t* test was used to determine the impact of the intervention on the students’ understandings and attitudes about IPE.

**Results:**

Out of 800 invited students, 530 students responded to the pre-intervention RIPLS survey. A comparison of the pre-post intervention for the RIPLS subscales of teamwork and collaboration, professional identification, and professional roles showed a significant improvement of students’ attitudes with *p*-values 0.03, 0.00 and 0.00, respectively. All workshop moderators scored a median of 4 or 5 to the essential elements of IPE during intervention except for a median of 3 for group dynamics.

**Conclusion:**

The present data, derived from the application of a brief online educational intervention, underpins the readiness and positive attitudes of undergraduate medical students towards IPE. The positive impact of online intervention necessitates the development of a structured and unified IPE curriculum to enhance the receptiveness and application of IPE in the medical field.

**Supplementary Information:**

The online version contains supplementary material available at 10.1186/s12909-021-02900-8.

## Introduction

Interprofessional education (IPE) embraces faculty and students from two or more health professions where they jointly learn in a collaborative environment [1]. An overlapping terminology is Interprofessional Learning (IPL) which, as coined by Reeves and Freeth, refers to a learning process that can result from the interaction between members of two or more professions [2]. However, IPL is not necessarily an outcome of IPE since it can occur spontaneously in workplace among members of different disciplines [3, 4]. From the viewpoint of the clinical environment, the best practice model of IPE is the interprofessional education and collaboration where a patient-centered focus is considered in a climate of multi-disciplinary healthcare system [5]. The primary objective of IPE is to produce healthcare professionals who can work together in an integrated patient-centered team to improve health-care outcomes and satisfaction [6].

The vast expansion of medical specialties and subspecialties, together with an unprecedented sophistication of skills required in each specialty, have created a genuine need for IPE in today’s clinical practice. In fact, the World Health Organization (WHO) has endorsed a deliberate achievement of IPL to improve patient health and safety (l*earning together to work together for health*) [7]. Such emphasis of the WHO on IPE restates its crucial role in healthcare professions. However, a well-structured program for embedding IPE in medical education and clinical practice is not available across the globe. In 2011, six professions joined together as an Interprofessional Education Collaborative Expert Panel to set core competencies for preparing healthcare professionals towards IPE [8]. These professions include six American Associations from Nursing, Osteopathic Medicine, Pharmacy, Dental Education Association, American Medical Colleges, and the Schools of Public Health. Since then, we have witnessed a rapid expansion of the IPE initiatives including the development of several curricula with horizontal and vertical integration [9, 10]. The role of IPE is becoming increasingly imperative in today’s medical education since it can significantly reduce the individual’s resistance towards interprofessional collaboration [11]. In an effort to assess the impact of IPE, Sanko et al., conducted a qualitative study on nursing and medical students by organizing a week - long simulation-based IPE course [12]. The researchers have reported that IPE and collaboration fostered an interactive and shared-decision mental model that helped to correct perceived misconceptions and to overcome barriers across healthcare professionals.

Unfortunately, a remaining challenge for the expected readiness of IPE is the limited implementation of the concept of multidisciplinary healthcare in most clinical settings worldwide. This factor minimizes the opportunities for effective collaboration among healthcare professions [13]. Additionally, there is a lack of a unified and validated educational framework that can embed IPE among healthcare professionals from different disciplines. A crucial hindrance to the smooth inculcation of IPE modules into the existing medical curricula is class scheduling [14]. Creating an IPE calendar that can cater diverse learners’ needs is a challenge and sometimes not possible.

Over the passage of time, the consistent and wider adoption of IPE can reshape how future clinicians and healthcare systems would apply interprofessional collaboration in routine clinical practice. However, during and after the COVID-19 pandemic, little is known about the effectiveness of online interventions and education of IPE that can comprehensively reciprocate the face-to-face educational models. The online courses are shown to be more flexible for students and faculty with busy schedules and can be remotely accessed with impactful teaching and learning activities [15]. Unfortunately, literature does not provide evidence of the impact of an IPE intervention via a distance learning model on undergraduate or postgraduate medical and health sciences students.

Currently, there is a need to estimate the effectiveness and feasibility of different tools and methods to improve the delivery and implementation of IPE in the undergraduate and postgraduate medical training curricula. At the same time, the readiness of the learners from a range of medical and health sciences disciplines should be determined before the inclusion of IPE into the curricula. The present study aims to determine the impact of an online educational intervention of IPE on the undergraduate students from different medical and health colleges at the University of Sharjah (UoS), United Arab Emirates (UAE).

## Materials and methods

### Context and participants

We conducted a quasi-experimental study on the currently registered undergraduate students of the College of Medicine (CoM), College of Health Sciences (CHS), and the College of Dental Medicine (CDM) at the UoS, UAE between June and October 2020. A pre- and a post-test quasi-experimental design were used for the collection of data. The primary objective was to determine the impact of an online educational intervention of IPE on the recruited students and to compare the differences of attitudes and understandings between genders (to compare gender variations) and junior and senior (to compare the variations among students across years). Variations among students across years and genders would help us to develop a consensus among all groups of students for a common understanding and acceptance of IPE.

### Study settings and design

The CoM runs a 6-year MBBS program that adapts a problem-based, community oriented, and student-centered approach. The students have early clinical exposure and the program gradually enhances the integration of basic and clinical sciences with an emphasis on group work, clinical reasoning and community-based research. The CHS conducts seven undergraduate four-year programs that involve laboratory and clinical training. The programs offer an active learning environment for students using arrange of pedagogies such as team-based learning, problem-based learning and flipped classrooms. The CHS programs equip students with skills in conducting scientific research and prepare them to serve the patients and the scientific community immediately after their graduation. The CDM provides a five-year program of dental surgery, which provides holistic problem-based expertise through the integration of dental clinical practice and dental health sciences. Students are well trained to manage community-based health problems as well as individual patient care. Currently, there is no formal sturctured IPE curriculum or training in the participating medical colleges. However, some unstructured educational activities are done by some colleges.

We conducted this research in a pre-intervention, intervention, and a post-intervention phases. An anonymous online survey was used (Google® forms) to collect the participants’ responses in each phase. The online workshop was organized using Microsoft Teams® during the interventional phase of our study.

#### Pre-intervention phase

We used the 19 item-modified version of the Readiness for Interprofessional Learning Scale (RIPLS) in our study (Additional file 1: Appendix A) [16]. Using the feasibility sampling technique, we invited years 4 and 5 students from the colleges involved in this research. All other years’ students were excluded from this study. A total of 800 students were invited to participate in this phase. The RIPLS scale has three main components: a subscale of teamwork and collaboration (TC) from statements 1–9, a subscale of professional identity (PI) from statements 10–16, and a subscale of professional roles (PR) from statements 17–19. A five-point Likert’s scale including strongly agree, agree, undecided, disagree, and strongly disagree was used for all statements. In addition, the questionnaire included an open-ended qualitative question. Informed consents were obtained from all participants and the questionnaire was administered online 2 weeks prior to the intervention phase. This served as a benchmark to determine the baseline attitudes and readiness of the students about IPE.

#### Interventional phase

**Fig. 1:**
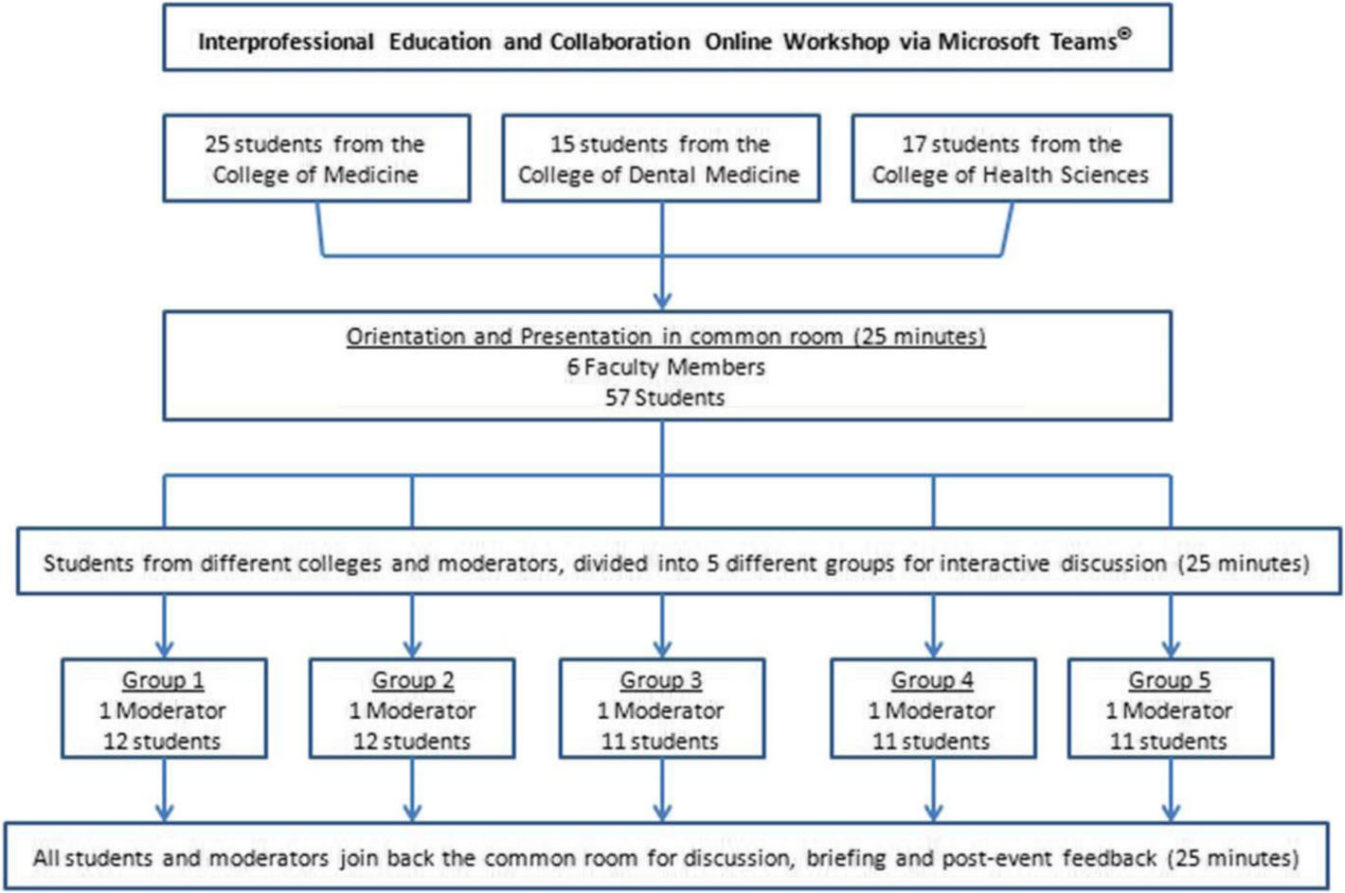
The layout of the online educational intervention about the interprofessional education and collaboration

During the interventional phase, an online workshop was organized via Microsoft Teams® where 55 students with equal representations from CoM, CHS, and CDM actively participated. Using the random sampling technique, we recruited 55 students who attended the pre-intervention phase, as they expressed their interest and willingness to participate in this phase. All those students who did not show interest to partcipitate in the intervention phase were excluded. The layout of the online workshop is illustrated in Fig. 1.

The intervention included a 20-min introduction and orientation of IPE by the principal investigator. The orientation part focused on the understanding, value and impact of IPE in patient care. Later, the participants were divided into five groups, each group had 11–12 students with comparable representations from CoM, CHS and CDM. Each group was assigned into a separate breakout room with a facilitator and a team organizer for technical support. Though there are no formal IPE courses in the medical and health sciences colleges of UoS, the facilitatros had sufficient experience in IPE as they were informally engaged in some IPE-based educational activities. In the breakout sessions, all five groups of students were instructed to simultaneously but separately solve a clinical scenario during a 30-min interactive case-based discussion session. One case scenario was used for all groups (Additional file 1: Appendix B). The case scenario described a patient with complex clinical conditions that require multidisciplinary care. The facilitators’ role was to oversee the discussion and observe interactivity and engagements among students. The facilitators encouraged the participants to engage and create an interprofessional climate by efficient communication and to secure a joint management plan. Finally, all participating students reconvened to the main online meeting room to share their verbal feedback and reflections in a 15-min session. Moderators from each group provided ratings of the online intervention using an online Google® Form tool. This tool contained ten statements about the degree of students’ engagement, group harmony and progression of consensus-building discussion sessions.

#### Post -intervention phase

Immediately after the intervention and case scenario discussions, the RIPLS questionnaire was administered to the same 55 students through Google Forms® to gather their post-intervention understandings about IPE by adopting purposive sampling method.

### Statistical analysis

The quantitative data were analysed using the Statistical Package for the Social Sciences (SPSS) Inc., version 20.0. We performed reliability analysis using Cronbach’s alpha and descriptive analysis by summary statistics, which includes mean, standard deviation, minimum and maximum values of three factors of RIPLS such as TC, PI and PR. This data was generated as clustered bar charts of frequencies. We also performed the independent *t* test to compare the responses of male and female students as well as junior and senior students. Finally, we performed a paired sample *t* test to determine the impact of the intervention on the students’ understanding and readiness about TC, PI and PR in IPE. A *p-*value of less than or equal to 0.05 was considered statistically significant.

## Results

During the pre-intervention phase, of 800 invitees, we received 530 complete responses (a response rate of 66%). The data included 183 students from CoM, 187 CHS, and 160 from CDM. There were 175 male and 355 female students and 304 junior (1st year to 3rd years) and 226 senior students (4th and 5th year). As reported in the survey, no student had any prior IPE experience.

The reliability of the RIPLS instrument is shown in Table 1, which confirms that all three RIPLS sub-scales of TC, PI, PR have greater cronbach’s alpha values than the cut point of 0.70. The descriptive analysis of the data showed that the sub-scale TC had the highest mean of 4.15, which indicates that students showed more agreement with the TC compared to other sub-scales.

**Table 1:** Reliability and descriptive statistics in this study (*n* = 530)

Sub-scale	Items	Reliability (α)	Minimum	Maximum	Mean	Std. Dev.
Teamwork and collaboration (TC)	S1-S9	0.87	1.00	5.00	4.15	0.60
Professional Identity (PI)	S10–16	0.79	1.00	5.00	3.29	0.49
Professional Roles (PR)	S17–19	0.77	1.00	5.00	3.08	0.75

**Fig. 2:**
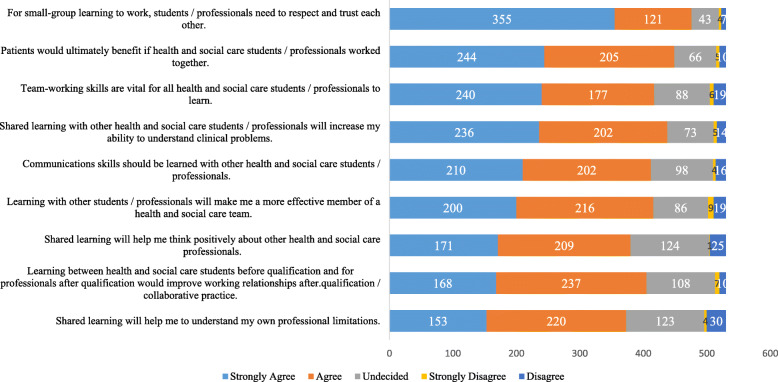
A clustered bar chart of the observed frquencies of responses to the subscale of teamwork and collaboration of the RIPLS inventory (*n* = 530)

**Fig. 3:**
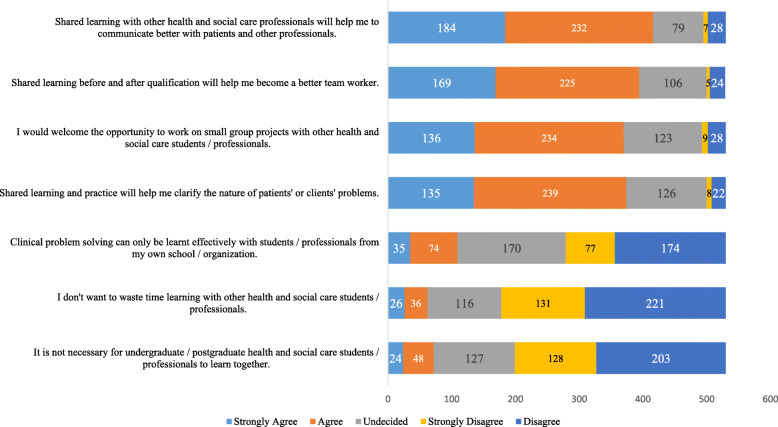
A clustered bar chart of the observed frequencies of responses to the subscale of the professional identity of the pre-intervention RIPLS inventory (*n* = 530)

**Fig. 4:**
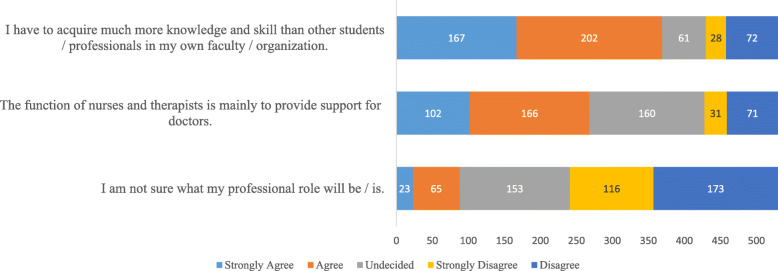
A clustered bar chart of the observed frequencies of responses to the subscale of professional role of the pre-intervention RIPLS inventory (*n* = 530)

The detailed responses to statements under three RIPLS subscales are shown in Figs. 2, 3 and 4. The observed frequencies of the responses to TC for statements 1–9 are shown in Fig. 2. We observed that the majority, 355/530 (63%), strongly agreed with the statement ‘*for small-group learning to work, students or professionals need to respect and trust each other’*. This finding indicates that the students showed their readiness for small group learning in IPE. Likewise, the responses to the other eight statements under subscale TC are shown in Fig. 2.

The observed frequencies to the subscale PI of the RIPLS inventory (statements 10–16) are shown in Fig. 3. In this category, a majority, 184/530 (35%), strongly agreed with the statement ‘*shared learning with other health and social care professionals will help me to communicate better with patients and other professionals*. Interestingly, we noticed that the students showed strong disagreement with the statement, *‘I don’t want to waste time learning with other health and social care students/professionals’* with the highest 221/530 (42%) disagreement rate.

The observed frequencies of responses to the subscale PR for statements 17–19 of the RIPLS inventory are shown in Fig. 4. The strongest agreement, 167/530 (42%), was observed for the statement, ‘*the function of nurses and therapists is mainly to provide support for doctors.* The students showed strong disagreement, 173/530 (33%), to the statement ‘I am not sure what my professional role will be/is’.

The results of the independent sample *t* test for the comparison of the responses between male and female students to the RIPLS inventory is shown in Table 2. First, from the entire cohort, we found significant differences in opinions about subscales of TC and PR. Female students’ readiness (4.21 ± 0.54) for TC was significantly higher than male students’ readiness (3.95 ± 0.77). On the other hand, male students’ readiness (3.22 ± 0.81) for PR was significantly higher than female students (3.05 ± 0.73). Second, for subgroup analysis of the three colleges, we observed that readiness for TC in female students from the CoM was significantly higher than male students. The readiness for PI was higher in male students from the CHS. We did not find a significant difference in opinions between male and female students from CDM.

**Table 2:** Comparison of pre-intervention responses between male and female students from all colleges (*n* = 530)

Sample	Scale	Gender (n)	Mean ± Std. Dev.	***t*** value	***p*** value
**Overall**	TC	Male (175)	3.95 ± 0.77	−4.04	0.00
		Female (355)	4.21 ± 0.54		
	PI	Male (175)	3.35 ± 0.59	1.45	0.15
		Female (355)	3.27 ± 0.45		
	PR	Male (175)	3.22 ± 0.81	2.14	0.03
		Female (355)	3.05 ± 0.73		
**College of Medicine**	TC	Male (63)	3.88 ± 0.84	−2.90	0.00
		Female (120)	4.20 ± 0.59		
	PI	Male (63)	3.31 ± 0.66	0.48	0.63
		Female (120)	3.27 ± 0.53		
	PR	Male (63)	3.26 ± 0.83	0.84	0.40
		Female (120)	3.15 ± 0.79		
**College of Health Sciences**	TC	Male (53)	4.00 ± 0.61	−1.83	0.07
		Female (134)	4.22 ± 0.49		
	PI	Male (53)	3.62 ± 0.56	3.76	0.00
		Female (134)	3.26 ± 0.39		
	PR	Male (53)	3.25 ± 0.78	1.82	0.07
		Female (134)	2.96 ± 0.68		
**College of Dental Medicine**	TC	Male (59)	4.01 ± 0.78	−1.73	0.09
		Female (101)	4.20 ± 0.52		
	PI	Male (59)	3.25 ± 0.47	−0.49	0.63
		Female (101)	3.29 ± 0.46		
	PR	Male (59)	3.10 ± 0.77	0.22	0.83
		Female (101)	3.07 ± 0.72		

Using the independent sample *t* test, the comparison of responses to the subscale of TC, PI and PR between junior and senior students from all three colleges is outlined in Table 3. Overall, we found significant differences only for PR where senior students’ readiness (3.15 ± 0.71) was significantly higher than the junior students’ readiness (3.95 ± 0.77). For the subgroup analysis of colleges, a distinct pattern was recorded between junior and senior students of the CDM. However, we did not find any significant difference among students of CoM and CHS.

**Table 3:** Comparison of pre-intervention responses between junior and senior students from all colleges in this study (*n* = 530)

Sample	Scale	Student (n)	Mean ± Std. Dev.	***t*** value	***p*** value
**Overall**	TC	Junior (304)	4.16 ± 0.53	0.52	0.60
		Senior (226)	4.13 ± 0.75		
	PI	Junior (304)	3.30 ± 0.44	0.02	0.98
		Senior (226)	3.29 ± 0.45		
	PR	Junior (304)	2.98 ± 0.86	2.23	0.03
		Senior (226)	3.15 ± 0.71		
**College of Medicine**	TC	Junior (107)	4.13 ± 0.57	0.79	0.43
		Senior (76)	4.05 ± 0.84		
	PI	Junior (107)	3.34 ± 0.49	1.04	0.30
		Senior (76)	3.25 ± 0.66		
	PR	Junior (107)	3.30 ± 0.69	1.69	0.09
		Senior (76)	3.09 ± 0.93		
**College of Health Sciences**	TC	Junior (106)	4.20 ± 0.45	0.86	0.39
		Senior (81)	4.10 ± 0.88		
	PI	Junior (106)	3.30 ± 0.38	−0.12	0.91
		Senior (81)	3.31 ± 0.72		
	PR	Junior (106)	3.01 ± 0.70	1.33	0.18
		Senior (81)	2.79 ± 0.69		
**College of Dental Medicine**	TC	Junior (91)	4.13 ± 0.58	−1.01	0.31
		Senior (69)	4.23 ± 0.56		
	PI	Junior (91)	3.23 ± 0.50	−1.35	0.18
		Senior (69)	3.34 ± 0.43		
	PR	Junior (91) Senior (69)	2.90 ± 0.83 3.21 ± 0.68	2.32	0.02

A comparison of responses of students during pre-post workshop using a paired *t* test is outlined in Table 4. Using the RIPLS inventory, the readiness of all students for IPE significantly improved after intervention for all subscales. However, the acceptance of the subscale PI improved more significantly by intervention with a highest mean difference of 0.41 than TC and PR subscales.

**Table 4:** A pre-post workshop analysis of the responses by the participating students (*n* = 55)

Scale	Survey	Mean ± Std. Dev.	Mean difference	***t*** value	***p*** value
Teamwork and collaboration (TC)	Pre-workshop	1.32 ± 0.43	−0.30	−2.25	0.03
	Post-workshop	1.62 ± 0.59			
Professional Identity (PI)	Pre-workshop	2.19 ± 0.54	−0.41	−3.82	0.00
	Post-workshop	2.60 ± 0.36			
Professional Roles (PR)	Pre-workshop	2.47 ± 0.46	−0.35	−3.12	0.00
	Post-workshop	2.82 ± 0.27			

Finally, the ratings by the facilitators of the online intervention workshop are provided in Table 5. For most observations, the facilitators ranked a high median of 4. However, a low median of 3.5 was recorded for the statement, ‘*there were sufficient contributions to patient care from different disciplines*. This showed that the facilitators were either undecided or partially agreed with the statement. For the statement, ‘*the group recognized when the team was not functioning well’* the facilitators remained undecided with a median of 3.

**Table 5:** Ratings of workshop’s facilitators for the online educational intervention

Statement	Mean	Median	Minimum	Maximum
1. Participants effectively worked in an interdisciplinary team.	4.28	4.00	3	5
2. Participants treated team members as colleagues.	3.72	4.00	2	5
3. There were sufficient contributions to patient care from different disciplines.	3.33	3.50	2	5
4. Disagreements among students were handled effectively.	3.78	4.00	2	5
5. The group was able to develop an interdisciplinary management plan.	3.94	4.00	3	5
6. The group recognized when the team was not functioning well.	3.89	4.00	3	5
7. The group dynamics was appropriate.	3.22	3.00	2	5
8. There was interest and engagement of participants from all disciplines.	3.39	4.00	2	5
9. There was effective and clear communication among participants.	3.11	4.00	1	5
10. Interprofessional education and learning increased confidence of students in clinical decision-making.	4.39	4.00	3	5

## Discussion

This quasi-experimental study has shown a substantial baseline pre-intervention understanding and readiness of the undergraduate medical, dental and health sciences students about IPE. The online educational intervention enhanced the readiness and receptiveness of the representative cohort in all subscales of the RIPLS inventory. Though we did not prefer online intervention for IPE, the restirctions by COVID-19 pandemic left us with no other choice except for an online workshop. Nevertheless, our study signifies the impact of an online intervention with remarkable teamwork and collaboration. The recruited cohort of students from a range of disciplines virtually met and worked in small groups for the first time during their undergraduate training. Finally, the post-intervention data analysis showed a significantly positive impact of the online workshop where small groups of students from diverse disciplines envisaged to collaboratively solve a scenario under the supervision of multidisciplinary facilitators. The mechanics and harmony of the learning environment, observed in this study, paves the way to introducing further collaborative interprofessional training in medical colleges. Likewise, the effectiveness of the online intervention encourages the utilization of distance learning method in future teaching of IPE courses.

In this study, students scored a pre-intervention highest mean for the subscale TC. Literature has argued that IPE takes place when different healthcare professionals work collaboratively with patients, relatives, caregivers, and societies to deliver the highest level of care [17]. Historically, medical and health sciences students are taught in silos. Once they graduate, they suddenly face a different working environment of clinical practice where they anticipate uncertainties in their new roles, show poor interprofessional communication, less respect for colleagues’ roles, and misconception of one another’s scope of practice [18]. All such challenges endanger patient safety and dissatisfaction towards working in teams. To circumvent this misconception, Hallin et al., have evaluated the medical, nursing, physiotherapy and occupational therapy students’ attitudes after attending a clinical teamwork training [19]. The study has shown that all students’ groups reported increased perceived knowledge of the other three professions. Additionally, the authors have proposed that the intervention had positively contributed to the understanding of communication and teamwork for the improved healthcare outcomes. Such active interventions for IPE courses during undergraduate training carry great promise in breaking professional barriers and in harmonizing the collaborative practice.

In our study, w have observed that, during the pre-intervention phase, a great majority of respondents strongly agreed with the RIPLS statement ‘*for small-group learning to work, students or professionals need to respect and trust each other’*. This resonates well with our study design where the online educational workshop was structured to adapt small group learning in a case-based discussion format. Our post-intervention analysis has shown a significant improvement of the students’ attitudes towards IPE by working in small groups. A plethora of published reports have endorsed the impactful role of small group learning in the medical field [20, 21]. From a different perspective, we have observed that 42% students showed strong disagreement with the statement, *‘I don’t want to waste time learning with other health and social care students / professionals*. Again, this indicates the readiness of the participating cohort of students for further development and implementation of IPE in their curriculum.

For the PI subscale of the RIPLS inventory, we have noticed a highest agreement of students about the role of effective communications among the professionals, patients, families, and community stakeholders in IPE. The overarching concept of compassionate patient care relies on effective communication, which aims at developing meaningful and purposeful relationships among patients and professionals [22]. Nevertheless, a professionally competent physician with poor communication skills can create misunderstanding, judgemental errors, and dissatisfaction among patients resulting in overall substandard clinical care. Therefore, enhancing communication skills of undergraduate medical students within an IPE context is essential [23].

In our study, a pre-intervention analysis of gender differences towards three subscales of the RIPLS inventory has highlighted a better understanding of female students for TC than male students. In contrast, male students’ readiness for PR was significantly higher than female students. Similarly, the subgroup analysis of the students’ perceptions from three colleges has shown significantly more readiness for TC among the CoM female students than males. By and large, we did not find a significant difference in opinions of male and female students from CHS and CDM. In the study by Falk et al., the authors have deduced that male students from different programs were slightly, but significantly, less positive than female students during an interprofessional training ward course [24]. The authors have argued that gender should be considered during interprofessional clinical training as well as during the development of IPE curricula. Using simulation sessions in an IPE climate, Tamás et al., have reported that female students showed better communication skills and teamwork than their male counterparts [25]. Furthermore, the investigators have maintained that simulation scenarios were more inspirational and motivating for female participants. These findings of gender differences in attitudes towards IPE closely articulate with our findings, which may reflect a stereotype difference in the approach towards IPE and collaborative practice. Literature has not provided a logical justification for such gender differences. However, in institutions with a dominant gender representation, such findings may be of significance for the implementation of the IPE curriculum.

Overall, we have found that senior students’ readiness to accept PR was significantly higher than junior students. This finding is at par with the evolutionary phases and progressive maturity that medical students attain during their educational journey. MacDonald et al., have investigated the degree of knowledge about the role of others in an IPE atmosphere and have introduced a set of behavioural indicators that can facilitate educators in evaluating students in IPE courses [26]. These behavioural indicators for the interprofessional competency include a recognition of professional territories when the scope of one’s profession ends and another’s begins, resolving misconceptions, and respecting others’ roles during collaborative efforts. Recognition of distinct professional roles and identities among a team of diverse healthcare professionals is also crucial for an ideal environment of IPE [27].

The moderators in our educational intervention awarded a high median of 4 to most statements. This reasserts the value of integration, harmony, and collaborative learning among students from different medical disciplines. Finally, a comparative analysis of the responses from pre-post workshop for the subscales of TC, PI and PR has reported a significant improvement in understandings and attitudes of students towards IPE. Similar studies have shown a long term [28] as well as a short term [29] positive impact of IPE interventions on medical students and healthcare professionals. Regrettably, the positive impact cannot be sustained longitudinally. This is attributed to several obstacles and challenges that educators face during the implementation phase of IPE due to diverse disciplines, overcrowded timetables [30], inadequate resources [31], faculty resistance to the change [32], diverse teaching styles [33] and administrative hurdles [34]. Some of these barriers can be overcome by regular faculty development programs [35] and by developing a curricular framework including representations from all medical and health sciences faculties [36]. Finally, regularly spaced online intervention about IPEs, for instance one workshop in each semester, will have a sustained impact. This mechanism will also intensify the depth of professional relations among healthcare students that will provide a foundation for their long-term collaborative education and practice. These technology-enhanced educational programs are well supported by the use of social media for education which will closely resonate with the learning styles of the the current students of the z generation [37].

### Study limitations

We conducted this study during the peak of the COVID-19 pandemic when all restrictions of social distancing, partial lockdown were enforced in the UAE. This necessitated the use of an online application of Microsoft Teams®, which may have deterred physical interactions among students and faculty. Additionally, faculty and moderators from different colleges were new to each other and had only virtual introductions during the online interventional workshop. Lastly, we used RIPLS questionnaire which is an old validated tool to capture the participants’ readiness and understandings about IPE and might not have holistically explored the research area in the modern world.

## Conclusion

This study reports a positive impact of an interactive online educational intervention on the attitudes and readiness of the medical, dental and health sciences students towards IPE. Though the pre-intervention readiness of the students was encouraging, the post-intervention scores were significantly higher. These findings provide insightful data to medical educators for developing a standard IPE curriculum in the Gulf region.

## Supplementary Information

**Additional file 1.**

## Data Availability

The questionnaire and the workshop case scenario are attached as appendices A and B, respectively. Any ddditional data can be provided on request. The corresponding author, Prof. Salman Yousuf Guraya, will provide additional data, if requested.
