# Supplementary material for: The impact of an online intervention on the medical, dental and health sciences students about interprofessional education; a quasi-experimental study
Source: BMC Med Educ. 2021 Aug 30;21:457. doi: 10.1186/s12909-021-02900-8 (PMC8403513; doi:10.1186/s12909-021-02900-8)
Supplement: Supplementary file 1 — Additional file 1. [file 12909_2021_2900_MOESM1_ESM.docx]

**Appendix A. Study Questionnaire (Readiness for Interprofessional Learning Scale)**

Interprofessional education (IPE) reflects a situation where professionals learn with, about and from each other. In medical field, it is imperative to work together in a team for interdisciplinary workflow that aims at patient safety and better outcomes.

This survey aims to capture perceptions of participants towards interprofessional education. The data will help us to draft an IPE framework that can be applied across medical disciplines. This survey will take approximately 10 minutes.

Thank you in advance for completing this survey.

**A. College (Please pick the college you are enrolled in):**

1. College of Medicine
2. College of Health Sciences
3. College of Dentistry
4. College of Science

**B. Gender**: a. Male b. Female

**C. Year of Schooling:** a. 1 b. 2 c. 3 d. 4 e. 5

**D. Have you had previous experience of Interprofessional Learning?**

1. Yes b. No c. Do not know

**If Yes: (give a very brief statement of what was IPE learning and the impact of it you had):**

______________________________________________________________________________________________________________________________________________________________________________________________________________________________________________________________________________________________________________________________________________

E. **Please answer the following in a 5- Point Likert Scale.**

| Sl. No | Statements | Strongly Agree | Agree | Undecided | Disagree | Strongly Disagree |
| --- | --- | --- | --- | --- | --- | --- |
| 1 | For small-group learning to work, students / professionals need to respect and trust each other. |  |  |  |  |  |
| 2 | Patients would ultimately benefit if health and social care students / professionals worked together. |  |  |  |  |  |
| 3 | Team-working skills are vital for all health and social care students / professionals to learn. |  |  |  |  |  |
| 4 | Shared learning with other health and social care students / professionals will increase my ability to understand clinical problems. |  |  |  |  |  |
| 5 | Communications skills should be learned with other health and social care students / professionals. |  |  |  |  |  |
| 6 | Learning with other students / professionals will make me a more effective member of a health and social care team. |  |  |  |  |  |
| 7 | Shared learning will help me think positively about other health and social care professionals. |  |  |  |  |  |
| 8 | Learning between health and social care students before qualification and for professionals after qualification would improve working relationships after.qualification / collaborative practice. |  |  |  |  |  |
| 9 | Shared learning will help me to understand my own professional limitations. |  |  |  |  |  |
| 10 | Shared learning with other health and social care professionals will help me to communicate better with patients and other professionals. |  |  |  |  |  |
| 11 | Shared learning before and after qualification will help me become a better team worker. |  |  |  |  |  |
| 12 | I would welcome the opportunity to work on small group projects with other health and social care students / professionals. |  |  |  |  |  |
| 13 | Shared learning and practice will help me clarify the nature of patients' or clients' problems. |  |  |  |  |  |
| 14 | Clinical problem solving can only be learnt effectively with students / professionals from my own school / organization. |  |  |  |  |  |
| 15 | I don't want to waste time learning with other health and social care students / professionals. |  |  |  |  |  |
| 16 | It is not necessary for undergraduate / postgraduate health and social care students / professionals to learn together. |  |  |  |  |  |
| 17 | I have to acquire much more knowledge and skill than other students / professionals in my own faculty / organization. |  |  |  |  |  |
| 18 | The function of nurses and therapists is mainly to provide support for doctors. |  |  |  |  |  |
| 19 | I am not sure what my professional role will be / is. |  |  |  |  |  |
| **If you have any further comments regarding interprofessional education please enter them below** | | | | | | |

Thank you once again for completing this survey. The data will provide us with an understanding of the influence of the Interprofessional Collaborative Practice program that we are facilitating or implementing. The original RIPLS survey has been adapted for use by Latrobe Community Health Service & the Health & Social care Interprofessional Network (HSIN), Victoria – August 2009.

**Appendix B**

**CASE SCENARIO**

A 37-year-old woman, 14-week primigravida, presents to the emergency room with a sudden onset of lower abdominal pain for 6 hours. Three days ago, she had a drainage of a tooth abscess and she is taking antibiotics since then. The patient also complains of a feeling of nausea and diarrhoea with loose stools. On examination, the patient has a rapid pulse and a low-grade temperature. On examination, there is generalized abdominal tenderness and bowel are audible with increased frequency.

1. **Discuss the case among group for 10 minutes and bring consensus about this scenario** **(15 minutes)**
2. **Then address the following concerns (10 minutes)**
3. List the medical disciplines that should be included in this patient care? Give justification.
4. Do you identify any error in this patient’s management?
5. How interprofessional practice can improve this patient’s care?

***NB. The moderator will facilitate this group work and will enter the discussion for facilitation and continuity of discussion only.***
